# Lectures upon Diseases of the Heart

**Published:** 1889-06

**Authors:** 


### BOOK NOTICES.

LECTURES UPON THE DISEASES OF THE HEART. By E. M. Hale, M. D. Third edition. Pages, 478. Price, $3.25. Hahnemann Publishing House, Philadelphia. 1889.

The second edition of Dr. Hale's work was issued about six years ago; this new edition contains much new matter, both in pathology and materia medica. There are articles upon such subjects as "The Relations of Abnormal States of the Heart to Abnormal Conditions of Other Parts of the Body," "Oertel's Treatment of Weak Heart," "Is the American Heart Wearing Out?" "Cardiæsthenia," "The Effects of Tobacco on the Heart." In the line of therapeutics attention is called to such drugs as Adonis, Barium, Cereus, Convallaria, Caffeine, Nerein, Spartein, Strophanthus. The therapeutic measures recommended are, of course, chiefly such as are used by Dr. Hale, and are of little use to the strict Hahnemannian, who knows of no such drugs as "heart remedies." The Repertory, by Dr. E. R. Snader, is the most valuable portion of the work.

